# New local health officials: health departments’ newest leaders

**DOI:** 10.3389/fpubh.2025.1597909

**Published:** 2025-05-26

**Authors:** Chelsey Kirkland, Noah Yee Westfall, Krishna Patel, Timothy C. McCall, Jonathon P. Leider

**Affiliations:** ^1^Center for Public Health Systems, School of Public Health, University of Minnesota, Minneapolis, MN, United States; ^2^National Association of County and City Health Officials, Washington, DC, United States; ^3^Department of Clinical Research and Leadership, The George Washington University, Washington, DC, United States

**Keywords:** public health workforce, local health officials, PH WINS, NACCHO, descriptive

## Abstract

**Introduction:**

The purpose of this descriptive study was to compare the demographics of new and experienced local health officials (LHOs) and the rurality and size of the local health departments (LHDs) they serve.

**Materials and methods:**

Descriptive characteristics of new LHOs and experienced LHOs were compared between two national public health workforce datasets: the 2021 Public Health Workforce Interest and Needs Survey (PH WINS) and the 2022 National Association of County and City Health Officials (NACCHO) National Profile of Local Health Departments study (Profile). The 2022 Profile study was fielded from July through September 2022 from a population of 2,512 LHDs across the US. The 2021 PH WINS survey was fielded to a nationally representative sample of state health Agency-Central Offices and LHD staff. Descriptive characteristics were computed comparing new LHOs with experienced LHOs based on work status, age, gender, race/ethnicity, education level, rurality and size of the jurisdiction.

**Results:**

Approximately 30% of all LHOs are new (<2 years of experience) with no difference among jurisdiction sizes or rurality. Compared to experienced LHOs, a slightly greater proportion of new LHOs identified as Native American, Black, or Asian, and are younger.

**Discussion:**

Providing professional supports to new LHOs and addressing recruitment and retention challenges facing public health leadership can help ensure that the senior executive level of the public health workforce reflects the diverse and varied populations that it serves.

## 1 Introduction

There are roughly 3,300 local health departments (LHDs) across the United States (US) (1). In the US, the decentralized administration of public health activities is spread out between federal, state and local public health agencies (2). The US model is decentralized in two important ways - first public health is separated from health care (even primary health care and much of the clinical prevention delivered nationally) (2). Second, governmental agencies responsible for delivering public health are generally at the state or local level; centralization is decidedly lacking, as is common in countries around the world (2). This allows for some inefficiency in the organization of (clinical) preventive services, but a deeper view into how public health units, per se, work (3). This can inform countries or localities internationally attempting to grow their public health functions (2, 3).

The top executives that direct LHDs are a critical fixture to the public health system (46). While top executives have many different titles (e.g., health officer, medical director, health commissioner), they are most commonly known as local health officials (LHOs). Within each LHD, the LHO is the highest-ranking employee with administrative and managerial authority (2). LHOs have a broad scope of work that includes managing a government agency, setting goals and priorities, engaging with constituents on public health issues, working with a board of health, and communicating with other government agencies (4, 5). In the last few decades, LHO’s responsibilities have become more challenging due to extensive workforce shortages and turnover (6).

The public health workforce has experienced strained resources and high turnover in the past several decades, with significant decreases beginning in 2009 due in part to the Great Recession (6). These workforce challenges were observed at all levels of public health and were made more acute by the COVID-19 pandemic response (7). Public health executive (including LHO) workforce challenges are similar; about a quarter indicate an intent to leave their position in 1 year and over a third indicate an intent to retire within 5 years (8). When an LHO vacates their position, critical institutional knowledge is not always effectively transferred to the new LHO (6, 9). Notably, as of 2015, only about 40% of LHDs reported having a formal succession plan to ensure a smooth and effective transition of leadership (10).

New LHOs typically enter their position with robust professional knowledge, skills, and abilities (8); though some lack direct experience and institutional knowledge that is relevant for being an LHO at their specific health department. This is partly due to variations in governance structure, capacity, and needs of local LHDs, and populations served. Leadership development resources have demonstrated success in improving the retention of other profession leaders, suggesting new LHOs may benefit from tailored support and training, that could vary based on age and race/ethnicity (11, 12). For example, Kragt and Guenter (13) argue that older and more experienced leaders often need different types of supports compared to their younger, less experienced colleagues. Similarly, Irehill et al. (14) observed that younger leaders reported higher levels of burnout compared to older, more experienced counterparts, suggesting that new LHOs may benefit from professional support tailored to their age and experience. Both seasoned executives and young executives have professional needs, however they often differ depending on their age, level of experience, and stage of career.

Professional leaders that identify with racial/ethnic minority groups often experience professional and structural barriers to senior executive positions. For example, one study offers a general theoretical framework and cites empirical evidence for the ways mental health organizations frequently perpetuate structural racism (15). Other examples of barriers to senior leadership that racial minority leaders may experience include microaggressions (15), discrimination in hiring practices (16), lack of long-term mentor relationships (17), and limited professional development support for leaders of color (18). These barriers create additional challenges for minoritized leaders to move into senior leadership positions and reduce retention. Valuable professional supports to improve retention include mentorship opportunities for minoritized leaders (17) and affinity spaces (19). Experienced executive leaders can also help generate cultural capital for minoritized senior executive leaders that have less experience in their position by helping to “deconstruct the hidden rules of the game,” sharing knowledge of the mannerisms, dress and speech, and professional competencies that give credibility to their position as a new LHO (20, 21). One pilot program intending to improve the self-confidence and self-efficacy of nurse leaders from racial/ethnic minority groups successfully did so through mentorship and monthly workshops (17).

Given the evolving public health landscape and limited recent data on new LHOs, this descriptive study compares the demographics of new and experienced LHOs, examines the rurality and size of the LHDs they serve, and provides insights into the emerging new LHO workforce. Public health agencies can leverage these insights to tailor programs and resources that effectively support new LHOs in their role as the health department’s senior executive.

## 2 Materials and methods

### 2.1 Data

#### 2.1.1 The national profile of local health departments

The National Association of County and City Health Officials (NACCHO) conducts the National Profile of Local Health Departments (“Profile”) study every 3 years to develop a comprehensive nationwide description of infrastructure, workforce, and activities within local public health. The present study used the 2022 Profile, fielded from July through September 2022, to analyze a census of a population of 2,512 LHDs across the US; the response rate was 37.5%, yielding 942 LHDs in the final analytical sample, though all questions were optional. Detailed information about the methodology of the 2022 Profile study and the population of LHDs can be found elsewhere (1). Profile 2022 has a set of seven items directly asking about the LHD “top executive,” which is the LHO. Tenure was captured as the amount of time that the top executive had been in their position based on the start of their position and survey completion. Those without complete information on tenure (i.e., months and years) were excluded from the analysis. Any LHO with fewer than 2 years of tenure was classified as new, while two or more years of tenure was classified as an experienced top executive. Other items included the LHD top executive’s full-time status, gender, race, ethnicity, highest level of education, and degree specializations, as well as jurisdictional and organizational characteristics like rurality and size of health department. Rurality was defined using census data that was classified at the block group level. Size of LHD was based on the population served in the LHD jurisdiction. Small was <50,000 population; medium was 50,000–499,999 population; large was 500,000 + population.

#### 2.1.2 Public health workforce interests and needs survey

The de Beaumont Foundation conducts the he Public Health Workforce Interests and Needs Survey (PH WINS) is fielded every 3 years to assess the current governmental public health workforce’s demographics, job characteristics, training needs, engagement, wellbeing, and other critical factors (22). In 2021, the survey was open to all personnel employed by a state or LHD. The survey was fielded to a nationally representative sample of state health Agency-Central Offices and LHD staff and had 35% response rate [14,957 of 137,446 eligible respondents; see Robins et al. (23) for further detail on the methods]. LHO respondents were identified using the variable *Job Classification* response “Public Health Agency Director” or “Health Officer” as well as the variable *Setting* not including the response of “state health agency central office.” Other items included the LHD top executive’s full-time status, gender, race, ethnicity, and highest level of education. Survey respondents provided tenure to the nearest year. Respondents without a tenure response were excluded from the analysis. Any LHO with fewer than 2 years of tenure was classified as new, while two or more years of tenure was classified as an experienced top executive. Other items included the LHD top executive’s full-time status, gender, race, ethnicity, highest level of education, and degree specializations, as well as jurisdictional and organizational characteristics like rurality and size of health department.

Profile and PH WINS used the same definitions for jurisdictional and organizational characteristics, however, small LHDs were excluded from PH WINS analysis based on PH WINS sampling design.

### 2.2 Statistical analyses

#### 2.2.1 NACCHO profile

Statistical analyses were conducted in Stata 18 (StataCorp, 2023). Analyses used post-stratification weighting based on seven different sizes of population served to provide nationally representative estimates; finite population correction was utilized. Descriptive statistics were run for each item, with cross tabulations of each across subgroup of new/experienced top executive (i.e., <2 years experience, ≥2 years experience).

#### 2.2.2 PH WINS

Statistical analyses of the 2021 PH WINS Survey were conducted in Stata 18 (StataCorp, 2023). Descriptive statistics were run for each item, with cross tabulations of each across subgroup of new/experienced top executive (i.e., <2 years experience, ≥2 years experience). Analyses were weighted using national balanced repeated replication weights on the local sampling frame to adjust variance for the complex sampling design and non-response (20) to be representative of LHDs with at least 25 employees in the country by the 10 Health and Human Services regions and jurisdiction population size (> 25,000 and ≤250,000 or >250,000). The initial sample included 26,933 respondents from 439 LHDs with more than 25 staff whose population served was above 25,000; the estimates computed from the analytical sample are not representative of smaller LHDs. Ultimately, the analytical sample was defined as a respondent who identified as a “public health agency director” or a “health officer” and was not from a setting defined as “state health agency central office” and included 297 respondents. Statistical significance was not assessed in the analyses for either Profile or PH WINS.

## 3 Results

The descriptive statistics, including the subgroup analyses, for both Profile and PH WINS are presented in Table 1. Overall, new LHOs with under 2 years of tenure tended to be younger, with both PH WINS and Profile results finding that a higher proportion of new LHOs were 40 years or under (Profile: 34%; PH WINS: 41%) compared to experienced LHOs that were 40 years or under (Profile: 11%; PH WINS: 21%). Additionally, new LHOs tended to have less higher education (i.e., Master or Doctoral degree) and identified as white less often than their experienced counterparts. While Profile did not showcase this difference, PH WINS data suggested that new LHOs tended to be more Hispanic/Latino and more likely to work part-time compared to experienced LHOs.

**Table 1:** Characteristics of LHD top executives, experienced compared to new.

Top executive characteristic	Profile		PH WINS	
	Experienced %	New %	Experienced %	New %
Work status (*n* = 851; *n* = 297)				
Part-time	4%	4%	4%	8%
Full-time	96%	96%	96%	92%
Age (*n* = 833; *n* = 278)				
40 years and under	11%	34%	21%	41%
Over 40 years old	89%	66%	79%	59%
Gender (*n* = 851; *n* = 293)				
Male	29%	31%	34%	34%
Female	70%	68%	65%	66%
Prefer not to respond/Another Identity	1%	1%	2%	0%
Race (*n* = 851; *n* = 283)				
American Indian or Alaska native	1%	2%	1%	0%
Asian	2%	2%	7%	10%
African American or Black	5%	6%	15%	16%
Native Hawaiian or Pacific islander	N/A	N/A	0%	2%
White	93%	87%	73%	68%
Other	1%	0%	N/A	N/A
Prefer not to answer	1%	3%	N/A	N/A
Ethnicity (*n* = 851; *n* = 293)				
Not Hispanic or Latino	96%	93%	85%	71%
Hispanic or Latino	3%	3%	15%	29%
Prefer not to answer	1%	4%	N/A	N/A
Highest Degree (*n* = 839; *n* = 293)				
Associates	6%	12%	2%	2%
Bachelors	27%	29%	21%	35%
Masters	53%	46%	53%	48%
Doctorate	15%	14%	24%	16%

Some totals do not equal to 100% as items were asked as select all that apply or due to rounding; Data presented are weighted to the study population of 2,512 for Profile, but sample n for each item is also provided just for reference.

Across both urban and rural areas, approximately 70% (Profile: 70, 70% respectively; PH WINS: 72, 70% respectively) of LHDs have experienced LHOs and 30% have new LHOs (indicating about 30% LHO turnover), which is similar to all LHDs. While there does not appear to be a difference between urban/rural jurisdictions, the size of the jurisdiction based on population served shows some differences. Smaller LHDs reported a higher proportion of new LHOs (Profile: 34%) compared to medium and large LHDs (Profile: 24, 29%, respectively; PH WINS: 27, 37%, respectively) (Table 2).

**Table 2:** Rurality and size of LHDs with new and experienced LHOs.

Data source	Rurality		Size of LHD*		
	Rural %	Urban %	Small %	Medium %	Large %
Profile					
Experienced LHOs	70%	70%	66%	76%	71%
New LHOs	30%	30%	34%	24%	29%
PH WINS					
Experienced LHOs	70%	72%	–	73%	63%
New LHOs	30%	28%	–	27%	37%

Percentages rounded to the nearest whole percent. *Size of LHD is based on the population served in the LHD jurisdiction. Small is <50,000 population; medium is 50,000–499,999 population; large is 500,000 + population. Small LHDs were excluded based on PH WINS sampling design.

## 4 Discussion

This study described the demographic characteristics of new versus experienced LHOs and the rurality and size of the jurisdictions they serve. Overall, about 30% of all LHOs were new (<2 years of experience). Additionally, compared to experienced LHOs, new LHOs were younger and a slightly greater proportion of new LHOs identified as Native American (Profile: 2%; PH WINS: 0%), Black (Profile: 6%; PH WINS: 16%), or Asian (Profile: 2%; PH WINS: 10%). NACCHO Profile data did not indicate a difference between the proportion of new LHOs serving in rural versus urban communities and only a slight difference regarding the size of the LHD (i.e., small, medium, large). The following discussion highlights potential factors that contributed to the turnover among public health leadership and identifies strategies to improve recruitment and retention of new LHOs, and public health leadership overall.

### 4.1 Recruitment and retention challenges

Overall, data suggests that public health leaders are spending less time in their positions. The public health sector has a history of strained resources and high turnover among health officials in the past several decades and particular decreases since the early 2000s (6). Since the early 2000s the average tenure for public health executives has decreased from around 9 years, to around 7 years (1). A jump in turnover was similarly experienced during the COVID-19 pandemic response (7). In 2021 about a quarter of public health executives indicated an intent to leave their positions within the next year and over a third indicated an intent to retire within 5 years, often citing COVID-19, work overload/burnout, and stress as reasons for leaving (8). Aligning with strained resources, about 30% of public health executive respondents indicated pay as a reason for leaving their position (8). Leadership turnover can be costly for organizations, including financial strains and losses to institutional knowledge (24). However, as demonstrated by our findings, the changes in employment for LHOs can also be an opportunity for a younger and more racially diverse cohort of individuals to enter senior public health leadership positions. While we did not assess trends over time in this study, Profile data showed that between 2019 and 2022 the proportion of new LHOs grew from 21% in 2019 to 30% in 2022 (1). Succession planning could ease LHO turnover burden as it provides a formal or informal process for “intentionally identifying, developing, and retaining individuals for future management and leadership roles” (25). However, as of 2015, only about 40% of LHDs reported having a succession plan (10) and no subsequent literature was found on the prevalence of succession plans. Health departments that indicated high concern for staff retention had 2.5 times higher odds of having a succession plan (10). Accordingly, a succession plan may improve the retention of new LHOs.

### 4.2 New LHO diversity

The public health workforce can better serve their community when it is representative of the racial and ethnic composition of the served populations (26, 27). Research has shown that organizations with diverse leadership tend to have a more diverse workforce composition (24). We found that the percentage of new LHOs identifying as American Indian/Alaska Native, Hispanic, Asian, and African American/Black was higher compared to experienced LHOs. When addressing issues of turnover for racial/ethnic minority executive leaders in particular, Ursel et al. (28) have cautioned against treating minority executive leaders as a monolith. For example, the authors’ found that within the racial/ethnic minority categorization, Asian and Hispanic chief executive officers (CEOs) actually had lower turnover compared to White CEOs, and Black CEOs had similar turnover compared to White CEOs. Enhanced diversity among public health leaders is a crucial first step for increasing representation within LHDs. In turn, achieving greater diversity in leadership requires cultivating a more diverse overall workforce. One program endeavoring to do this was Public Health AmeriCorps, which placed members (often young workers) in public health settings and provide them with support and encouragement to enter public health careers (29). However, Public Health AmeriCorps and other fellowships have been shuttered in recent months.

To support diverse folks entering public health leadership, supports (e.g., leadership development, networking opportunities, mentoring, and skills development) and additional pathways into senior-level positions are also needed for public health professionals from varying racial and ethnic backgrounds. For example, the Association of State and Territorial Health Officials’ (ASTHO) Diverse Executives Leading in Public Health (DELPH) program provides professional development to state and local public health leaders from a variety of backgrounds with skill building, coaching, and professional networking opportunities (30).

Health departments have started to express their commitment to diversity, equity, and inclusion (DEI) (31). This commitment, despite current political climates, is expected to continue because of DEI’s prominence within the Public Health Accreditation Board’s standards and measures (32). However, institutional commitment alone is not sufficient for achieving substantive changes to the overall racial/ethnic composition of public health leadership. For example, the Urban Institute has catalogued some of the most important characteristics of 28 leadership development programs (including curriculum features, program length, individual/cohort participant model, and presence of collective leadership components), across a range of professional sectors (nonprofits, community based organizations, public/private businesses), that specifically focus on equity and leadership development for racial/ethnic minority leaders (18). The Minority Political Leadership Institute (MPLI) is an example of a program that demonstrates the current work being done to build leadership capacity for racial/ethnic group leaders in a variety of sectors including higher education, corporate, nonprofit, public agencies, and faith-based organizations. This program works toward actionable policy objectives that support leaders of color (33).

### 4.3 New LHO rurality

Previous literature has found that rural LHD leaders were less likely than urban LHD leaders to report an intent to leave (34). However, this was not observed in our findings - the size and rurality of LHDs served by new LHOs were the same as those served by experienced LHOs. This lack of difference could be due to significant recruitment and retention challenges facing both urban and rural health departments alike (34, 35). While new LHOs may not be serving a higher proportion of one type of LHD (e.g., small, rural), they likely need tailored support due to differences in the needs and capacities of various sizes and rurality of LHDs.

### 4.4 Preparing new public health leaders for success

With LHOs having a 30% turnover within both urban and rural LHDs, there is an opportunity to focus on the emerging needs of senior public health leadership workforce nationwide. There are many educational pathways into public health leadership, even from leaders with educational training outside of formal public health. For example, one recent study found that governmental public health professionals with a non-public health master’s degree were just as likely to hold a supervisory role as those with a formal master’s in public health (MPH) degree, though this analysis was not specific to LHOs (36). New LHOs may be able to learn some of the skills of successfully leading a health department through leadership development and training opportunities. There is evidence that leadership development programs are an effective strategy at improving the knowledge, skills and performance of professional leaders, according to a 2017 meta-analysis of 335 leadership training studies (37). However, younger leadership, especially with more new LHOs under the age of 40, likely have different needs than previous generations, signifying a need for LHDs to consider how their recruitment and retention efforts align with those of younger generations (38). Helm-Murtagh and Erwin (38) identified seven core themes needed for developing a new generation of public health leaders: building trust/accountability, promoting partnerships, connecting with healthcare systems, building information systems, systems and strategic thinking, recognizing structural racism as a primary driver of health inequities, and maintaining resilience and self-care. Other crucial public health competencies needed to ensure an effective and responsive local health department include epidemiology, disease surveillance (39) and community engagement (40). Public health executives have also indicated training needs in budget and financial management, policy engagement and systems/strategic thinking (8). Lastly, new LHOs should be prepared to lead in political environments where their legitimacy is challenged and their policies face heightened scrutiny (41).

We echo the recent call from Helm-Muragh and Erwin (38) to improve professional development resources for public health leaders. National organizations representing the interests of the public health workforce (e.g., NACCHO and ASTHO) can play an important role in helping to develop and implement leadership resources and reduce barriers for new LHOs (38, 42). In the past, the Centers for Disease Control and Prevention’s (CDC) Public Health Leadership Institute graduated 806 senior leaders between 1992 and 2006 (42), providing valuable professional development resources through retreats, conference calls, webinars, and coaching (42). The public health leadership institute also improved skills, knowledge, and professional networks for program participants (42). Then, from 2008 to 2012 NACCHO offered the Survive and Thrive training program that provided new LHOs with tailored training, peer learning, networking opportunities, and coaching from experienced health officials (43). Currently, the Diverse Executives Leading in Public Health (DELPH) program provides professional development to mid-senior level public health leaders (30) and the New to Public Health Residency Program (N2PH) administered through the University of Wisconsin provides emerging governmental public health leaders with curriculum components, and mentorship (44). While these programs help prepare public health leaders (30, 42–44), more are needed to adequately train the incoming cohort of new LHOs with the skills that they need to be successful.

## 5 Limitations

Though this study used large, nationally representative datasets, it does have limitations. First, the two datasets were cross-sectional and conducted at different times, so data could not be aggregated and we could not draw conclusions about what happened between the two administration dates. Although we observed about 30% turnover among LHOs in a single year, we did not assess turnover across multiple years to identify turnover trends. Additionally, there are potential discrepancies in the identification of new LHOs between the two datasets. In the NACCHO Profile, information regarding LHOs was captured in a unique section. In PH WINS, LHOs were extrapolated from respondents who identified as an “agency director” or “health officer,” creating potential incongruence between who was categorized as an LHO between the two datasets. Furthermore, while we observed differences between new and experienced LHOs in our data, we do not know if the differences are statistically significant. Though survey weights were used and non-response was taken into account, PH WINS did not collect enough data from small health departments and the findings may not be generalizable to other small health departments. This lack of generalizability may also explain why the PH WINS data suggested a more diverse workforce than Profile data. This analysis was limited by staff and population size owing to how it was fielded by the data owners themselves, not an analytic choice on our part. We find the concern plausible; work from others has looked at this specific question and has shown that the lack of representativeness is most present in staff demographics, educational attainment, and training needs (45). But the scope of difference is modest and we do not believe it materially impacted our results (45). Finally, this study was specific to the US public health system and may not necessarily generalize to other countries.

## 6 Conclusion

This descriptive study used PH WINS and Profile data to describe the demographic characteristics of new versus experienced LHOs and the rurality and size of the jurisdictions they serve. Overall, about a third of all LHOs are new, and compared to experienced LHOs, new LHOs are younger and a greater proportion identify with racial/ethnic minority groups. While turnover can be costly for organizations and create uncertainty, changes in leadership can also open pathways for a younger and more racially diverse cohort of individuals to enter into senior public health leadership positions. Health departments can also take an active role in helping to recruit new LHOs from varied backgrounds and orient new LHOs into their position by leveraging succession planning. While programs exist to prepare public health leaders, more are needed to adequately train the incoming cohort of new LHOs with the skills that they need to be successful. Professional resources can help prepare new LHOs for their new position and improve retention, especially for leaders that are currently underrepresented. Addressing the core recruitment and retention challenges facing local public health leadership and providing tailored professional support is essential for maintaining an effective and responsive public health infrastructure by ensuring the local public health workforce reflects the varied populations that it serves.

## Data Availability

The data analyzed in this study is subject to the following licenses/restrictions: not all of the data cited in this article are publicly available because of the nature of the authors’ data use agreement. Requests to access these datasets should be directed to PH WINS: www.debeaumont.org/phwins/2021-findings/ NACCHO profile: www.naccho.org/resources/lhd-research/national-profile-of-local-health-departments.
